# OAM light propagation through tissue

**DOI:** 10.1038/s41598-021-82033-6

**Published:** 2021-01-28

**Authors:** Netanel Biton, Judy Kupferman, Shlomi Arnon

**Affiliations:** Electrical and Computer Engineering Department, Ben-Gurion University of the Negev, P.O Box 653, IL84105 Beer-Sheva, Israel

**Keywords:** Biotechnology, Engineering, Optics and photonics

## Abstract

A major challenge in use of the optical spectrum for communication and imaging applications is the scattering of light as it passes through diffuse media. Recent studies indicate that light beams with orbital angular momentum (OAM) can penetrate deeper through diffuse media than simple Gaussian beams. To the best knowledge of the authors, in this paper we describe for the first time an experiment examining transmission of OAM beams through biological tissue with thickness of up to a few centimeters, and for OAM modes reaching up to 20. Our results indicate that OAM beams do indeed show a higher transmittance relative to Gaussian beams, and that the greater the OAM, the higher the transmittance also up to 20, Our results extend measured results to highly multi scattering media and indicate that at 2.6 cm tissue thickness for OAM of order 20, we measure nearly 30% more power in comparison to a Gaussian beam. In addition, we develop a mathematical model describing the improved permeability. This work shows that OAM beams can be a valuable contribution to optical wireless communication (OWC) for medical implants, optical biological imaging, as well as recent innovative applications of medical diagnosis.

## Introduction

Ever since it was shown in 1992 that Laguerre Gauss beams carry orbital angular momentum (OAM) beams, many studies have investigated this phenomenon and have brought a new era in optical research^1,2^. The properties of OAM have been explored in many areas such as wired/wireless communication^3–6^, imaging and medical diagnosis^7,8^, particle traps^9,10^, and quantum encryption^11,12^. New methods for creating OAM beams have been explored and their propagation through diffuse media has been studied. Recent works have focused on the beam structure for different orders of OAM after passing through diffuse media^7,8,13^. As a beam passes through diffuse media, the light is scattered the polarization becomes random and the beam's power decreases^14^.

Studies of OAM propagation have treated transmission through multi scattering media for communication purposes, including transmission through atmosphere, desert environment and underwater^6,12,15–18^. In such studies, transmittance was examined as a function of the topological charge, $\ell$ , representing the order of OAM. Some experiments conducted in an underwater communication system have shown that there is no major advantage in an increasing the order of the OAM^17^. In contrast, studies examining the effect of increasing beam order on beam penetration through biological tissue have shown that increasing order improves beam penetration. In recent years, the connection between attenuation and OAM has been investigated in the context of biology and medicine^7,8,13,19–21^. Studies that have used numerical methods that model this problem have shown that increasing the topological charge does improve permeability and increases the contrast of the outgoing beam^22–24^. These results were consistent with results of other experiments on OAM transmission^22^. In this work, we use relatively thicker biological tissue, reaching the cm range. In this way we verify and extend previous work on beam propagation through tissue in actual experiments at a macroscopic scale.

A relatively new field where study of OAM beam propagation through tissue can be relevant is optical wireless communication (OWC) with medical implants. The use of OWC with medical implants presents clear advantages over existing radio frequency (RF) communication with commonly used medical implants, such as: high data rate, lower power and cyber security^25–28^. Scattering of photons in the tissue leads to loss of signal power which lead to high bit-error-rate (BER). Advances in the field of OWC and new technologies have led to studies of better communication through tissue and have reduced the BER^29–32^. Increasing beam permeability can contribute to further reducing the BER, and increase of communication reliability. Obviously in the field of medical implants this is of paramount importance.

Beam permeability through tissue is influenced by wavelength and polarization, and if OAM too is effective this could be a turning point in transfer of communication with medical implants to the optical spectrum, development of non-invasive diagnostics and improvement of optical biopsy techniques. Therefore, understanding the effect of OAM beam order on tissue permeability can be of vital importance.

In this study, we examined the relation between the order of Laguerre Gauss (LG) beams and the permeability of a collimated beam through biological tissue. The aim, in contrast to earlier studies, was to understand the effect of increasing OAM on macroscopic samples of tissue. While earlier studies studied narrow slivers of tissue, in our work we used tissues of varying thickness as great as 2.6 cm. In addition, all tissue was taken from chicken breast, so that inhomogeneity of the medium played a relatively minor role, enabling us to focus solely on increase of OAM order. Therefore this work provides insight into the effect of OAM permeability on biological tissue and is a valuable indication of its potential value in medical applications.

**Figure 1:**
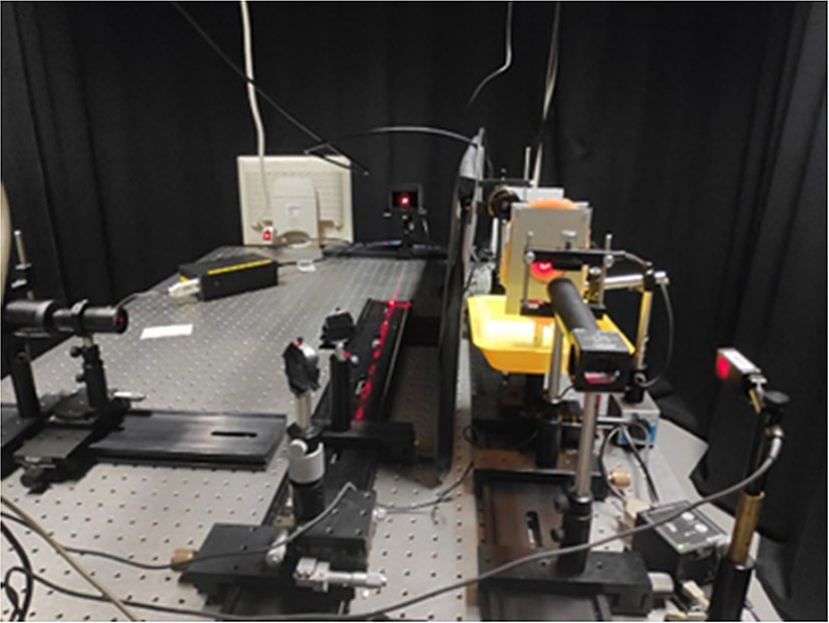
Photo of the experimental setup.

The main building block of the setup includes a laser source in the visible spectrum, Spatial Light Modulators (SLM), beam splitter, tissue sample (chicken breast) and power meter (see Fig. 1). The aim of the experiment was to examine the relation between the power transmittance and the order of the photon OAM. The results show that increasing the order of the LG beams increases the transmittance value, and this result is maintained with increased thickness of the medium.

The rest of the paper is as follows. In “Theory” the theory of OAM beams is outlined, along with the method of beam generation. “Experiment” describes the experiment itself. “Results and discussion” gives experimental results and discussion, and we finish with our conclusions.

## Theory

### Orbital angular momentum (OAM) of light

**Figure 2:**
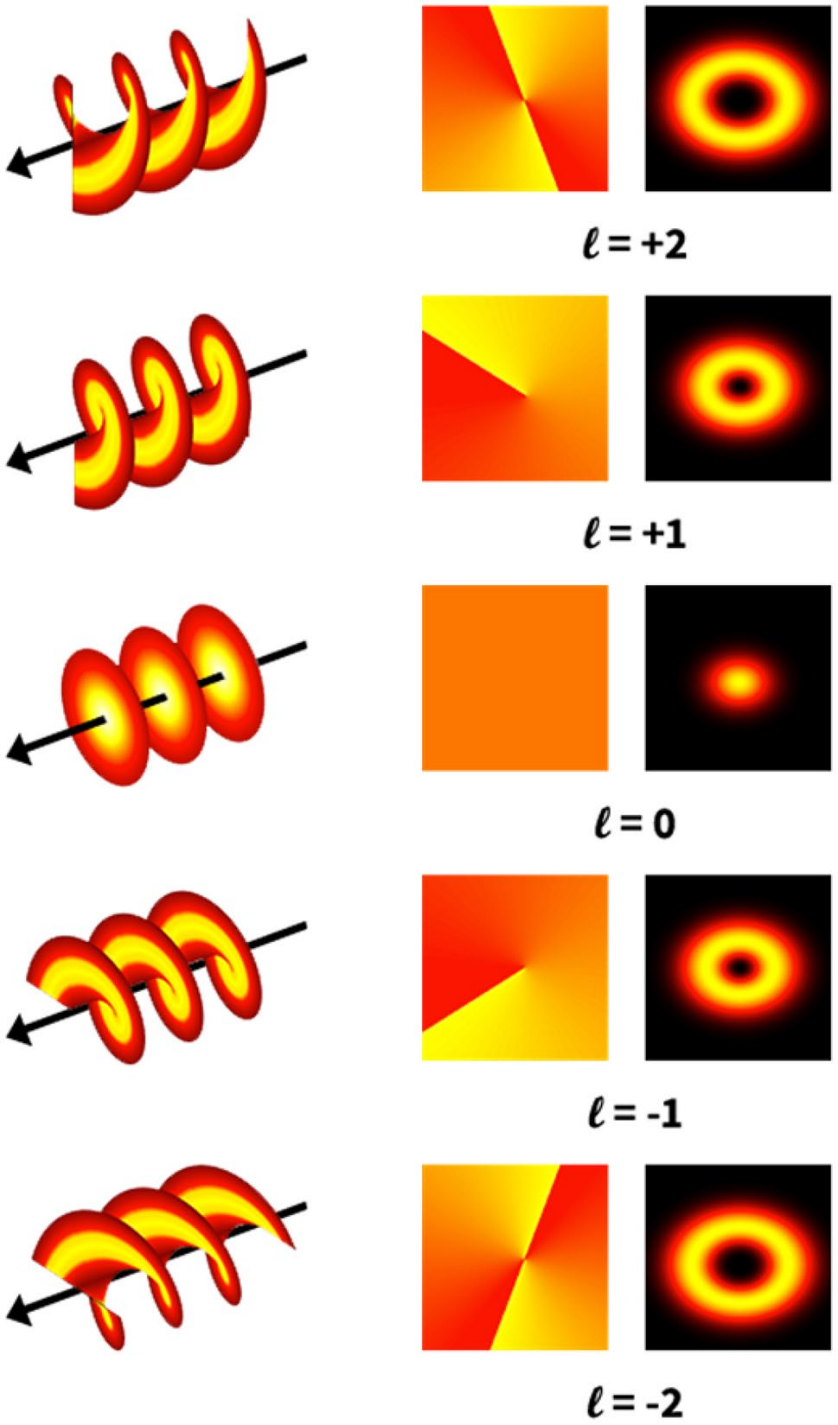
Different topological orders of OAM light beams. Columns left to right show the beam wave front, phase front and the intensity pattern.

The orbital angular momentum (OAM) of light determines the spatial distribution of the beam but not the polarization^1,2^. These beams have a helical or twisted phase front: the Poynting vector of the phase front is never parallel to the axis of propagation of the beam at any point but rather spirals around it in a helical form, while on the axis there is zero intensity, and therefore OAM beams are also called optical vortices. The wave function of an OAM beam includes a term ${e}^{i\ell \varnothing }$. Each photon carries angular momentum of L =|$\ell {\mathcal{\hslash }}$|, that is, an integer number times Planck’s constant. The different orders of OAM beams are represented by the integer $\ell$, where if the rotation direction of the phase front is clockwise $\ell$ is positive, and if the rotation is counterclockwise $\ell$ is negative (Fig. 2 left column). $\ell$ is called the ‘topological charge’, and it indicates the number of times the phase front completes a full cycle in one wavelength (Fig. 2 middle column). The intensity profile of an OAM beam is that of a ring and the larger $\ell$, the larger the radius of the ring (Fig. 2 right column). One type of OAM light beams are called Laguerre Gaussian (LG) laser modes. These LG modes provide a complete set of solutions to the paraxial Helmholtz equation in cylindrical coordinates, so that any OAM beam may be expressed as a linear combination of LG beams. They are convenient because they can be produced in several ways from Gaussian (G) beams. LG beams can have several concentric beams, which are given by an additional radial parameter $\mathcal{P}$ in the wave function. Since our interest is in the orbital angular momentum of the beam only, the beams used in the experiment all had $\mathcal{P}=0$. The wave function of such a beam is^33^:

1 $$E\left(r,\mathrm{\varnothing },z\right)={C}_{{\mathcal{P}},\ell }{\left(\frac{r\sqrt{2}}{w\left(z\right)}\right)}^{|\ell |}{L}_{{\mathcal{P}}}^{|\ell |}\left[\frac{2{r}^{2}}{{w\left(z\right)}^{2}}\right]{e}^{-\left(i\psi \left(z\right)+\frac{ik{r}^{2}}{2R\left(z\right)}+i\ell \phi \right)}$$

${C}_{p,l}$ is a normalization constant ${C}_{{\mathcal{P}},\ell }=\sqrt{\frac{2{\mathcal{P}}!}{\pi \left({\mathcal{P}}+\left|\ell \right|\right)!}}$ where $\mathcal{P}$ is radial coordinate index,$\ell$ is azimuthal coordinate index, $r$ is the radial distance from the center axis of the beam,$z$ is the axial distance from the beam's waist (the narrowest point in the beam). the function $w\left(z\right)$ describes the radius of the electric field amplitudes and written as $w\left(z\right)={w}_{0}\sqrt{\frac{{{z}_{r}}^{2}+{z}^{2}}{{{z}_{r}}^{2}}}$.${w}_{0}$ is the beam's waist radius,${z}_{r}$ is the Rayleigh range and is equal to ${z}_{r}=\frac{\pi {{w}_{0}}^{2}}{\lambda }$, $\lambda$ is the wavelength,${\mathrm{L}}_{\mathrm{p}}^{\mathrm{l}}[\mathrm{x}]$ are the generalized Laguerre polynomials, $\mathrm{i}$ is the imaginary unit,$\mathrm{k}$ is the wave number $\mathrm{k}=\frac{2\uppi }{\uplambda }$,$\mathrm{R}\left(\mathrm{z}\right)$ radius of curvature along the Z axis,$\uppsi (\mathrm{z})$ is the Gouy phase along the Z axis.

### Generation of OAM beams

OAM light beams can also be generated by various methods, for instance Q-Plates or cylindrical lenses^1,2^. We generated OAM light beams with various topological charges in the lab using a Spatial Light Modulator (SLM). The limit for highest possible topological charge is determined by resolution of the SLM^34^. SLM can modulate amplitude, phase, or polarization of light beams in space and time. For this experiment, a phase only Liquid Crystal on Silicon (LCOS) SLM was used, the Holoeye PLUTO-2 model. Phase only LCOS SLM allows creation of a light beam with a phase front that is predesigned by a computerized hologram. The SLM system enables simple creation of LG beams by projecting the spiral phase hologram. The Gaussian beam is incident on the SLM and it will receive the phase pattern according to the projected hologram. Only a certain percentage of the input beam will receive this phase pattern, and the percentage varies with different parameters such as device type, polarization, wavelength and more. The rest of the input beam is reflected as a Gaussian beam, and because the LCOS SLM is a reflective device, the result is a combination of LG and Gaussian beam. In order to separate the two beams we added a blazed hologram pattern to the spiral hologram pattern, giving a fork hologram. The blazed hologram actually moves the desired LG beam position to the first diffraction order, thus separating the SLM reflection Gaussian beam from the desired LG beam. The creation of the holograms and their screening for SLM is done through ‘HOLOYEYE SLM Pattern generator’ software.

### Light propagation through biological tissues

As a light beam propagates through tissue, the interaction with the cells in the tissue causes the photons to be absorbed or scattered^35^. The probability of photon absorption per unit length is given by the absorption coefficient, ${u}_{a}$. Scattering is elastic, and unlike absorption, where energy is converted to the other type of energy, the scattered photon is re-emitted with its original energy but with a probability to change its path. For biological tissue the re-emission of each photon is with high probability in the forward direction, but as a result of the multiplicity of scattering events a random propagation direction is obtained. The probability of photon scattering per unit length is given by ${u}_{s}$. The effect of multiple scattering events is taken into account by the reduced scattering coefficient ${u}_{s}^{\mathrm{^{\prime}}}={u}_{s}(1-\mathfrak{g})$, where $\mathfrak{g}$ is the anisotropy factor, and defines the degree of forward scattering; typically, the $\mathfrak{g}$ value of tissue ranges from 0.8 to 1. The attenuation coefficient for biological tissue is calculated as the sum of the absorption coefficient and the reduced scattering coefficient. Photons whose original direction is unchanged are called ballistic photons, from which information can be extracted for communication or imaging. The number of ballistic photons through a tissue is very small, determined by the type of tissue and the length of propagation through the tissue. Another type of photon from which information can be extracted are called snake photons, photons that are slightly scattered from the axis of propagation but still maintain a degree of coherence.

## Experiment

**Figure 3:**
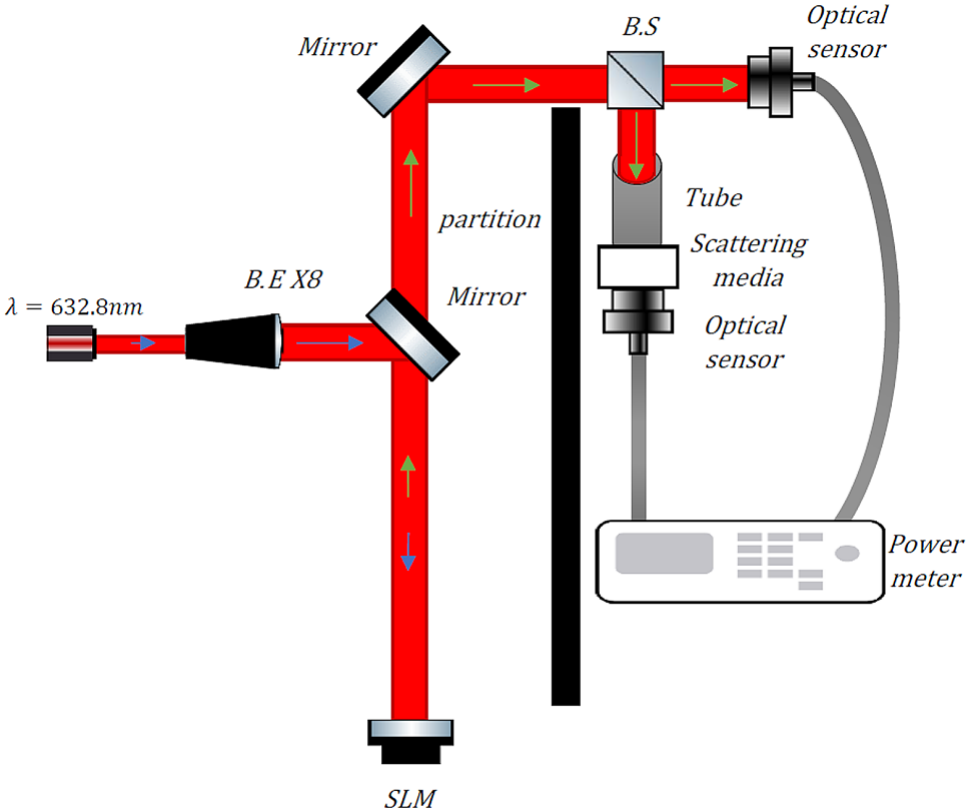
Schematic diagram of the optical setup. The source is a Gaussian (G) beam with a wavelength of 632.8 nm. The blue arrows indicate the direction of propagation of the G beam, while the green arrows indicate the direction of propagation of the LG beam.

The optical setup of the experiment is shown in Fig. 3. The setup includes a He–Ne laser with a power of 5mW and 632.8 nm wavelength as light source (Melles Griot 05-LHP-123-496). The laser-produced Gaussian beam enters a beam expander (B.E) with an 8 × magnification. The extended beam is shifted by an aluminum mirror to the center of the SLM crystal. When the Gaussian beam is incident on the center of the SLM, the reflected beam is an LG beam with the ℓ-order twin to the ℓ-order of the projected fork hologram to the device. The resulting LG beams are shifted by another aluminum mirror into the measurement area. The LG beams were then divided by 50:50 cubic beam splitter (B.S Thorlabs CCM1-BS013/M) into two beams. The first beam is used to measure power through the diffused media ${P}_{DM}$, and the second beam to measure power through free space ${P}_{FS}$. A black tube was connected at the first exit of the beam splitter to ensure that only the first diffraction order emerged on the other side and the rest of the diffraction order was blocked by the tube. On the other side of the tube, diffuse media was placed in the path of the first beam. To measure power ${P}_{DM}$, a measuring sensor (Ophir Optronics PD300-UV SENSOR) was placed adjacent and nearly touching the diffuse media. In the second beam path, and another measurement sensor was placed to measure the power ${P}_{FS}$. Both sensors were connected to a power meter with two measurement channels (Ophir Optronics Centauri Dual Channel), where the sensor measuring the ${P}_{DM}$ was connected to the first channel. The ${P}_{DM}$ measurement sensor was located on the propagation axis of the first beam. thus, almost all the ballistic and snake photons reached the sensor. The remaining photons of the beam were either scattered or absorbed in the media. Use of the two channels of the power meter allowed calculation of the transmission (T) at a narrow aperture in the direction of the beam propagation, and to be considered in the beam reflections, where the calculation is $T={P}_{Channel A}/{P}_{Channel B}$.

**Figure 4:**
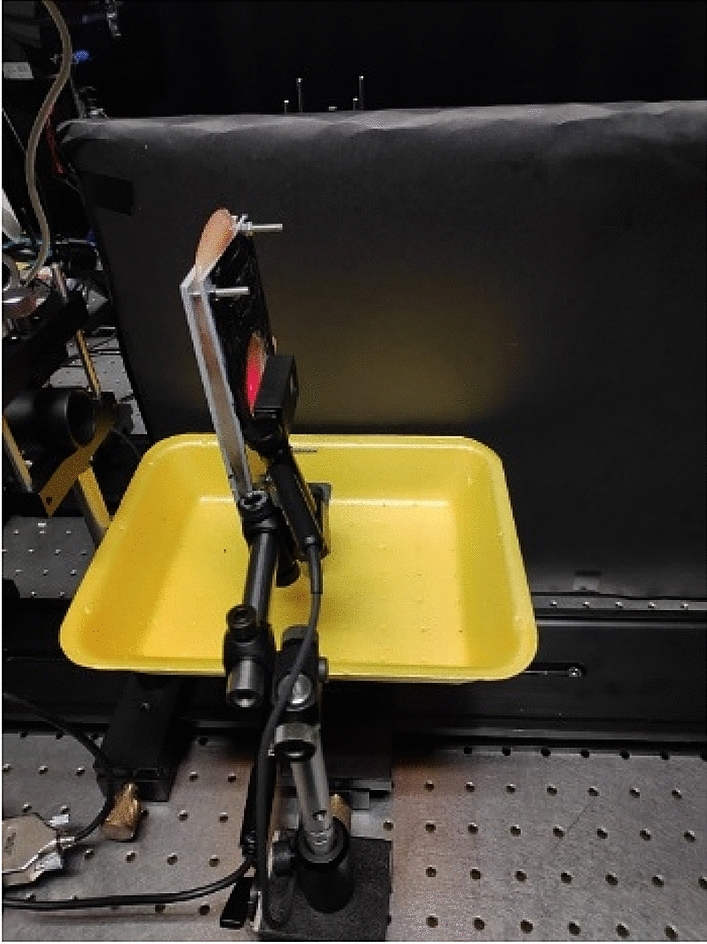
Photo of light propagating through the tissues.

As a tissue diffusing media, we used a fresh chicken breast, which was bought from the store in prepared slices, free from skin and fat. Chicken breast tissue, besides being readily available, is an extensively studied tissue, and its optical parameters are well known^36–42^. In this experiment, we wanted to examine the effect of increasing the *l* order of collimated beam on the permeability of the beam through tissue. We also wanted to test this effect for different thicknesses of tissue. The experiment measured transmission of 21 different orders of LG beams through four different thicknesses of chicken breast tissue (Fig. 4). In order to get different thickness of chicken breast, first we used one slice of 0.75 cm thick. Then we added another slice of 0.6 cm thick on top of the first slice, so total thickness of the slice is 1.35 cm. We continued the same process two more times until we reached a maximum thickness of 2.6 cm. The slices of the tissue are placed on a measuring stand, which is in fact two presses with aperture for the passage of the beam being examined. We chose to stop 2.6 cm thick to avoid measurements in the area that are close to the room noise. The LG beams had orders of ℓ ranging from 0 (Gaussian beam) to 20, where for each order we took 150 measurements of the transmission.

The noise in the measurement area was measured when the laser was turned off. Both of the sensors presented at least two orders of magnitude less than the measurements from the experiment. Therefore, the noise of the room can be neglected in comparison to signal amplitude. Measurements were made at room temperature of 21 ± 0.5 °C. Essential information on the experiment is summarized in Table 1.

**Table 1:** Essential information about the experiment.

Slice thickness	0.6–0.75 cm
Total thickness measured	0.75, 1.35, 2.0, 2.6 cm
Measurement stand length	8.4 cm
Measurement stand width	13.4 cm
Measurement stand aperture	4.7 cm
Sensor aperture	10 × 10mm
Room noise	1.35 nW

## Results and discussion

In this section, we present the measurement results of the transmission values (T) obtained for the different thicknesses of the chicken breast tissue and for the different $\ell$ values. By numerical calculations, we approximated the attenuation coefficient of chicken breast, and compared the results with other studies that investigated the issue.

**Figure 5:**
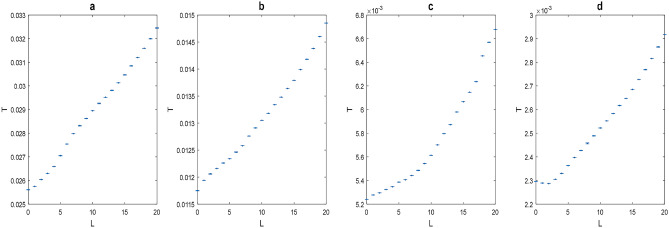
Results of measurements made for different thicknesses of tissue, where X axis represents topological order and Y axis transmission. Graph a is for tissue thickness of 0.75 cm, graph b for tissue thickness of 1.35 cm, graph c tissue thickness of 2.0 cm, graph d tissue thickness of 2.6 cm.

**Figure 6:**
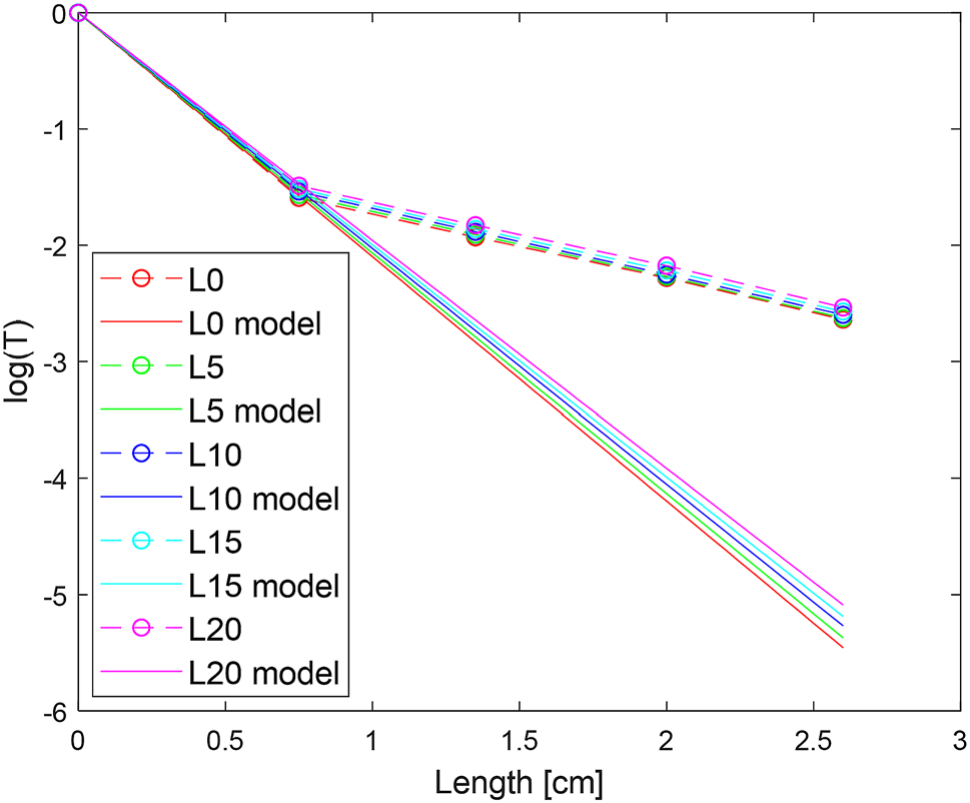
Transmission as a function of length; each line represents a different topological order of the beam. The solid lines represent the modeled transmission function, the dotted lines represent the measured values, one line for each topological order. The discrepancy between model and measured values is due to the fact that the model approximates transmission of ballistic photons only, and as thickness increases the number of snake photons increases as well.

We first examined the effect of the L order of the beam for 4 different thicknesses of chicken breast tissue (0.75, 1.35, 2.0, 2.6 cm). The results show that increasing the order of the LG beam improves the T value. Figure 5 shows the measurement results for different orders of LG beams, where the X axis represents the order of the LG beam, the Y axis the average of the T values of the measurements. Each graph represents a different thickness of tissue tested. It can be seen from the graphs that for high $\ell$ orders a higher transmission value is obtained, and this trend holds for all the thicknesses of tissue that have been tested. To better analyze the data, we focused on a number of representative $\ell$ orders. Figure 6 shows the measurement results of the representative orders of LG beams (l = 0, 5, 10, 15, 20), where the X axis represents the length of the tissue and Y axis the average results of the log(T) measurements, where the points in the graph are the values measured. For comparison, we normalized the results by those obtained from the Gaussian beam measurements ($\ell$ = 0), as shown in Table 2. The results show that for LG beams with the high orders of $\ell$ the T value shows an improvement. According to the BeerLambert law, the transmission of ballistic photons $(\mathrm{T{^{\prime}}})$ of a beam passing through media is equal to:

**Table 2:** Measurement results normalized by the measurement results of the Gaussian beam.

L	0.75 cm	1.35 cm	2.0 cm	2.6 cm	Transmittance (%)
5	105.62	105.02	102.76	102.91	104.08 ± 0.0145
10	113.04	111.06	107.09	109.84	110.26 ± 0.0249
15	118.976	117.36	115.74	116.94	117.26 ± 0.0134
20	126.70	126.38	127.41	127.04	126.89 ± 0.0044

Values are given in percentages relative to 100% of the transmitted Gaussian beam.

2 $${ I}_{out}={I}_{in}{e}^{-u\bullet d}\to \mathrm{T{^{\prime}}}=\frac{{I}_{out}}{{I}_{in}}={e}^{-u\bullet d}$$

where ${I}_{in}$ is the power of the input beam, ${I}_{out}$ the power of the output beam, $u$ is the attenuation of the media, equal to the sum of the absorption coefficient and the reduced scattering coefficient (See “Light propagation through biological tissues”). and $d$ is the propagation distance within the media.

To support our measurement result, using numerical calculations, we model an exponential curve for the measurements using the exponential function ${Ae}^{-b\bullet d}$, where A is a amplitude coefficient for the zero thickness (equals 1), $b$ is the estimated attenuation coefficient and $d$ is the propagation distance within the media. This model makes it possible to compare the estimated attenuation coefficient with the previous published results of experiments^36–42^. The optical coefficients of chicken breast are a topic that has been studied for many years, and there is a difference in the outcome in different studies. Despite the individual differences in study results, the studies do show that the reduced scattering coefficient is dominant in the attenuation. The causes of the variation in results can be due to several reasons such as method of measurement, device deviation and differences between the tissues examined. The various measurements show that for wavelengths of 630–633 nm the scattering coefficient can range from ${2.2-8.0\mathrm{cm}}^{-1}$. The value of the absorption coefficient can range from a wider range of ${0.03-1.2\mathrm{cm}}^{-1}$. Figure 6 shows the estimated model for each order of the beam versus the measurement values. The estimated attenuation value starts at $4.832{\mathrm{cm}}^{-1}$ for the Gaussian beam and decreases to $4.507{\mathrm{cm}}^{-1}$ for order 20.

This result corresponds to the coefficient of chicken breast attenuation according to various studies. The deviation between the curve and the measurements is relatively low, with the RMSE ranging from 0.0068 to 0.0084 (varies between orders). As can be seen from the graph, the discrepancy between the estimated value and the measured value increases as the tissue thickness increases. This result makes sense, because for the thicker tissues, the number of snake photons is greater than that of the ballistic photons reaching the detector, and therefore a higher value is measured. The numerical calculation results for each order are presented in Table 3.

**Table 3:** Results of numerical fit to function $T{^{\prime}}={e}^{-bd}$.

L	B (${\mathrm{cm}}^{-1}$)	R	RMSE
0	4.832	0.9998	0.006804
5	4.757	0.9997	0.007070
10	4.665	0.9998	0.007397
15	4.594	0.9998	0.007814
20	4.507	0.9997	0.008407

In our work, we have examined propagation in chicken breast. This differs from biological tissue explored in other work^7^. Chicken breast is defined as skeletal muscle tissue; the cells come from the base units of myofibril, that is, a muscle fibril, a basic rod-like unit of a muscle cell and homogeneous in fiber content^43,44^. This is more fairly homogeneous media. This allows us to draw firm conclusions about one parameter only: the effect of topological charge on penetration through homogeneous tissue. In^7^, for example, the authors investigated penetration through brain tissue, which is made up primarily of two broad classes of cells (neurons and glial cells)^45^. Neurons are complex cells containing a soma (cell body), dendrites and an axon; the soma is compact but the dendrites and axons are filaments. Glial cells come in a wide variety. Brain tissue is therefore not homogeneous, and at different depths the beam goes through very different media and the shape and density of cells vary depending on the region and the depth within the region^45,46^. Conversely, exploration of more homogeneous tissue enables us to clearly single out the effect of OAM.

## Conclusion

In this article, we investigated whether increasing the angular momentum of the light beam improves the beam permeability on the propagation axis. The results of our experiment show that the permeability for photons passed through a diffuse media for OAM beams is larger than that for Gaussian beams. OAM light beams with higher topological order showed higher transmission value. Measurements were made for different thicknesses of chicken breast, as tissue is a highly diffusing medium for the optic spectrum. The use of an optical measurement sensor allowed us to gain an indication of the photons exiting through the scattering media close to the propagation axis. That is, most of the photons measured are in a ballistic orbit or have a relatively small scattering angle and are in a snake orbit. The range of ballistic photons in the media is known to be very small, so most of the measured power is mainly photons with a low forward scattering angle. The forward scattering angle is mainly determined by the anisotropy factor and an increase in transmission value can indicate an increase in factor. The reason why the increase in topological charge increases the permeability of the beam is still under investigation. There are several hypotheses for this^8,24^ but no mathematical models or theoretical proofs.

Biological tissue is a strong scattering material. Therefore, there may be an additional effect due to scattering from the transverse interface of the tissue slices used here. However, this does not seem to affect the conclusions of our work, because clearly the increase in transmission with increasing order is the same for all thicknesses that we tested. Our different samples consisted of four slices, where the thinnest is only one slice and the thickest is four slices sandwiched together. However, they all show identical behavior: it is clear that the higher the topological order, the greater the emitted light. Therefore, we do not see a significant effect of the interfaces on the result. Nevertheless, this presents an interesting issue that should be explored in future work. Another interesting question for future work is to investigate the effect of change in sensor aperture, and its effect on improved information about the pattern of the scattering.

In conclusion, we have shown that light with orbital angular momentum has higher permeability through the biological tissue used in the experiment. Tissue samples were as thick as 2.6 cm, providing valuable insight into applications in real world situations. A wide range of OAM modes was tested, reaching as high as l = 20. Results clearly show that the higher the orbital angular momentum (the higher the topological charge), the greater the permeability. This suggests that use of light with OAM could indeed be a useful and exciting new tool for various applications of optical wireless communication, medical imaging and medical optical applications.
